# Doxorubicin Inhibits Phosphatidylserine Decarboxylase and Modifies Mitochondrial Membrane Composition in HeLa Cells

**DOI:** 10.3390/ijms21041317

**Published:** 2020-02-15

**Authors:** Nadège Bellance, Fabienne Furt, Su Melser, Claude Lalou, Didier Thoraval, Lilly Maneta-Peyret, Didier Lacombe, Patrick Moreau, Rodrigue Rossignol

**Affiliations:** 1 INSERM U1211, Rare Diseases: Genetic and Metabolism, F-33076 Bordeaux, France; 2 University of Bordeaux, F-33076 Bordeaux, France; 3 UMR 5200 CNRS, Membrane Biogenesis Laboratory, F-33140 Villenave d’Ornon, France

**Keywords:** phosphatidylserine decarboxylase, doxorubicin, bioenergetics, phosphatidylethanolamine

## Abstract

Doxorubicin (DXR) is a drug widely used in chemotherapy. Its mode of action is based on its intercalation properties, involving the inhibition of topoisomerase II. However, few studies have reported the mitochondrial effects of DXR while investigating cardiac toxicity induced by the treatment, mostly in pediatric cases. Here, we demonstrate that DXR alters the mitochondrial membrane composition associated with bioenergetic impairment and cell death in human cancer cells. The remodeling of the mitochondrial membrane was explained by phosphatidylserine decarboxylase (PSD) inhibition by DXR. PSD catalyzes phosphatidylethanolamine (PE) synthesis from phosphatidylserine (PS), and DXR altered the PS/PE ratio in the mitochondrial membrane. Moreover, we observed that DXR localized to the mitochondrial compartment and drug uptake was rapid. Evaluation of other topoisomerase II inhibitors did not show any impact on the mitochondrial membrane composition, indicating that the DXR effect was specific. Therefore, our findings revealed a side molecular target for DXR and PSD, potentially involved in DXR anti-cancer properties and the associated toxicity.

## 1. Introduction

Doxorubicin (DXR) is an anthracycline antibiotic, also known as Adriamycin, used for chemotherapy in cancer. Regarding its mode of action (MOA), a series of diverse cellular effects have been reported [1]. The more described MOA involves DXR interaction with DNA or topoisomerase II, and the consecutive inhibition of DNA synthesis. DXR was also proposed to induce reactive oxygen species (ROS) production and lipid peroxidation, although the molecular targets for this effect remain unclear [2]. Lastly, some effects of DXR on cell membranes have also been reported but remain poorly understood. Previous studies showed that DXR is able to decrease membrane fluidity and lipid organization in reconstituted biological membranes [3,4,5]. The contribution of these changes to DXR cytotoxicity is also not completely deciphered. However, these destabilizing membrane effects are the earliest dose-dependent alterations that occur in cells following drug exposure [3,6]. DXR was also shown to accumulate in the mitochondrial membrane compartment [7,8] and to sensitize cell death mechanisms [9,10]. In the mitochondrion, DXR binds to cardiolipins in an electrostatic manner [11,12,13]. However, the effect of DXR on mitochondrial membrane properties is not entirely elucidated, as no lipidomic evaluation of this compartment has been performed. Conversely, the importance of mitochondrial membrane composition and integrity for the proper functioning of oxidative phosphorylation is well established.

In mammalian cells, phosphatidylethanolamine (PE) can be synthesized at two compartments, in the endoplasmic reticulum (ER) by the Kennedy pathway via the cytidine diphosphate –ethanolamine (CDP-ethanolamine) route and in mitochondria by the phosphatidylserine decarboxylase (PSD). This last enzyme resides in the outer leaflet of the inner mitochondrial membrane and requires phosphatidylserine (PS) import from the ER [14]. Encoded by the *PISD* gene, the PSD proenzyme comprises α- and β-subunits separated from each other by an autocatalytic site. During the maturation process, the two subunits associate themselves to synthesize PE from PS by releasing CO_2_. Genetic alterations in the mitochondrial enzyme phosphatidylserine decarboxylase (PSD) triggers changes in the composition of the mitochondrial membrane and causes mitochondrial diseases characterized by severe bioenergetics dysfunction [15]. Tasseva et al. reported a reduced mitochondrial pool of PE in CHO *PISD* knockdown cells, characterized by a fragmented mitochondrial network [12]. Furthermore, PSD knockdown expression in the skeletal muscle of mice was responsible for the decrease in PE content and rise in PS with myopathy development [16]. Therefore, we hypothesized that PSD could play a role in the proposed mitochondrial membrane effects of DXR.

In the present study, the mitochondrial membrane composition of a cell line sensitive to DXR, such as HeLa, was analyzed by high-performance thin-layer chromatography. Then, we investigated the link between DXR with PE biosynthesis, mitochondrial membrane composition, energy metabolism, and finally, cell viability. We also revealed that a cell line less sensitive to DXR was characterized by a low basal PSD activity. This cell line synthesized PE from the CDP-ethanolamine branch of the Kennedy pathway.

Taken together, these findings are in accordance with a specific effect of DXR on cells with a high PSD activity that implies dependency on this enzyme for the mitochondrial pool of PE. Then, in chemotherapy, we can postulate that the sensitivity of DXR treatment is strongly related to the cell capacity for biosynthesis of PE.

## 2. Results

### 2.1. DXR is Incorporated Inside Mitochondria of HeLa Cells

**Figure 1:**
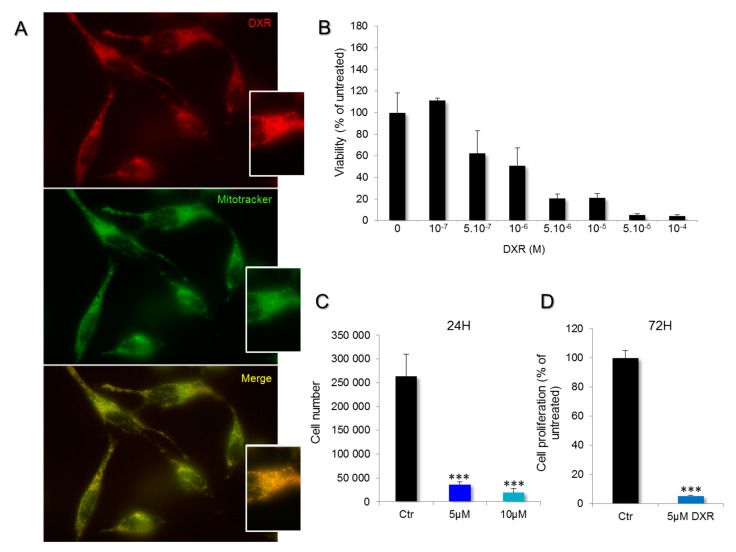
Incorporation of doxorubicin (DXR) in mitochondria of HeLa cells and impact on cell viability. (**A**) Living cells were treated with 100 nM Mitotracker Green and 5 μM DXR, for 30 and 10 min, respectively. The overlaps between mitochondria (green) and DXR (red) are shown in yellow. (**B**) The viability of HeLa cells was estimated after doxorubicin treatment for 24 h with a range of concentrations between 10^−7^ and 10^−4^ M. (**C**) The proliferation was measured, after 24 h with DXR (5, dark blue and 10 μM, light blue), by counting the cells in the presence of trypan blue, and it was compared to the condition without DXR, the control (Ctr, black). ***, *p* < 0.001; One-way ANOVA with multiple comparisons. (**D**) The effect of DXR on cell proliferation was also measured after 72 h of treatment and compared to the control untreated cells. ***, *p* < 0.001; unpaired t test, two-tailed.

DXR is usually described as a drug that can block DNA replication and transcription. It is an intercalating agent that inhibits topoisomerase activity, induces ROS production, and decreases energy production. To understand the mitochondrial effects of DXR, we first investigated its incorporation inside Hela cells. Doxorubicin uptake was analyzed by using fluorescence microscopy as DXR can emit red fluorescence. After 10 min of incubation with 5 μM DXR, the drug uptake was visualized. DXR entered and accumulated in the mitochondrial network (Figure 1A). The DXR signal was co-localized with that of the Mitotracker Green probe. The corresponding Pearson index was 0.97 ± 0.01 (*N* = 13). Then, the DXR effect on cell viability was estimated (Figure 1B). The viability of Hela cells was impaired after incubations with DXR higher than 0.5 μM. The calculated IC50 was 1.39 ± 0.17 μM. Accordingly, cell enumeration analysis showed that 13.62% of the cells remained after 24 h treatment with 5 μM DXR, and 7.33% for 10 μM (Figure 1C). These findings indicate that DXR enters the mitochondria of HeLa cells and that these cells are sensitive to DXR.

### 2.2. DXR Modifies the Mitochondrial Membrane Composition via PSD Pathway

**Figure 2:**
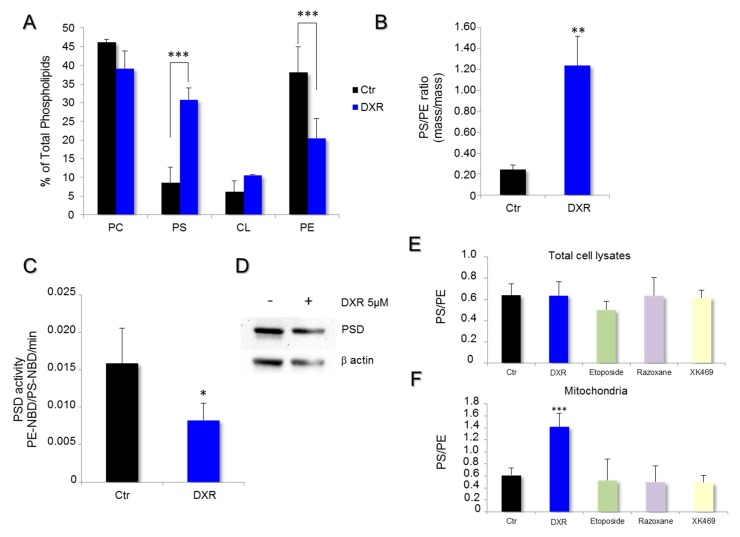
DXR modifies mitochondrial membrane composition of HeLa cells while inhibiting phosphatidylserine decarboxylase (PSD) activity. The effect of DXR on the phospholipid composition of mitochondrial membranes was analyzed by high-performance thin-layer chromatography. (**A**) The quantification of PC, phosphatidylcholine; PS, phosphatidylserine; CL, cardiolipin; and PE, phosphatidylethanolamine was performed by densitometry and expressed as a percentage of total phospholipids. The mitochondrial membrane composition obtained after 5 μM DXR treatment was compared to that in control conditions. ***, *p* < 0.001; Multiple *t* test of two-way ANOVA. (**B**) The PS/PE ratio was determined in HeLa cells, treated or untreated, with DXR at 5 μM (**, *p* < 0.01). (**C**) The rate of PSD activity was determined in vitro on isolated mitochondria using a fluorescent analog of phosphatidylserine, PS-NBD (black). The enzyme activity was also measured in the presence of DXR 5 μM (blue). The data are expressed in PE-NBD/PS-NBD ratio per minute. Each point represents the mean value ± SEM of *N* ≥ 3 experiments (*, *p* < 0.05). (**D**) The expression level of PSD was analyzed by Western blot on cell lysate treated by 5 μM DXR for 24 h, and compared to the untreated cells. The effect of topoisomerase inhibitors on PS/PE ratios was determined from (**E**) total cell lysates and the (**F**) isolated mitochondrial fraction. Cells were incubated with 5 μM DXR, 1.25 μM etoposide, 20 μM razoxane, or 10 μM XK469 for 24 h. Then, estimation of the PS/PE ratios was performed. The data are expressed as mean values ± SD with ***, *p* < 0.001.

To bring new insights to the cascade of events leading to HeLa cell death, we evaluated the contribution of the DXR destabilizing effect on mitochondrial membranes. The phospholipid composition of mitochondrial membranes was first determined by high-performance thin-layer chromatography and quantified by densitometry on cells treated with 5 μM doxorubicin for 72 h and compared to the untreated control conditions (Figure 2A). DXR altered the contents of PS and PE within the mitochondrial membranes, suggesting a molecular effect on PSD (Figure 2B). The drug induced an accumulation of PS and a reduction of PE, which is consistent with an inhibition of PSD activity. To validate this hypothesis, mitochondria isolated from HeLa cells were incubated with a fluorescent analog of phosphatidylserine (PS-NBD), and the rate of production of phosphatidylethanolamine-NBD (PE-NBD) was followed for 30 min. The results were expressed as variations per min of the ratio PE-NBD/PS-NBD. The enzyme activity was 0.0159 ± 0.0047 in the untreated control conditions, and 0.00827 ± 0.0023 in the presence of DXR. Thus, an inhibition of 47.95% was observed (*p* = 0.027) (Figure 2C). These results demonstrate that DXR treatment inhibits PSD with consecutive remodeling of the mitochondrial membranes.

To determine whether the DXR effect on membranes was only consecutive to PSD inhibition, the PS/PE ratio was estimated after treatment by different topoisomerase inhibitors, such as etoposide, razoxane, and XK469, for 24 h at 1.25, 20, and 10 μM, respectively. The concentrations used were known to avoid harming cell viability. For each drug used, adherent cells were collected and their lipids were extracted for PS/PE determination. Furthermore, no cell death was observed with etoposide, razoxane, and XK469 treatments. The measurement of the total cell lysates showed no difference for HeLa when compared to the control (Figure 2D). Regarding the mitochondrial membrane, the only modification observed was obtained with DXR, which increased the PS/PE ratio as compared to the control. 

### 2.3. Deleterious Impact of DXR Treatment on Energy Metabolism Depends on the Content of PE

**Figure 3:**
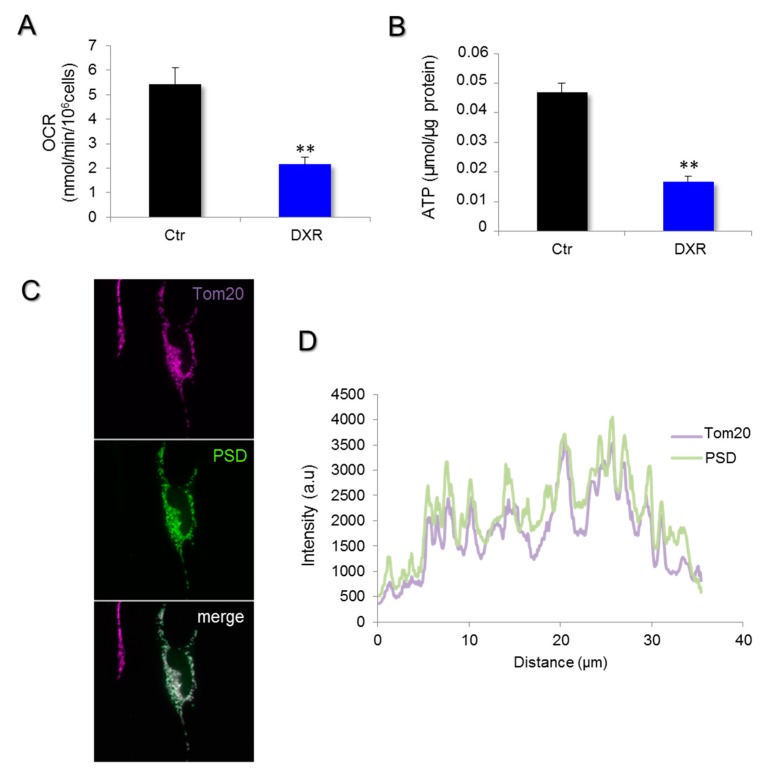
Doxorubicin inhibits mitochondrial respiration and reduces ATP content. (**A**) Mitochondrial respiration was measured in HeLa cells treated for 6 h with 5 μM DXR, and the endogenous rate of respiration was recorded. Data are expressed as mean values of oxygen consumption rates (OCRs) ± SEM, N = 5 experiments with *p* = 0.006 (**, *p* < 0.01). (**B**) The total ATP content of HeLa was determined by bioluminescence in cells treated or untreated with 5 μM doxorubicin for 24 h. The results are expressed in μmol/μg protein as mean values ± SEM. The difference was considered as significant when *p* < 0.05 (**, *p* < 0.01). (**C**) The PSD subcellular repartition was investigated by immunofluorescence on HeLa cells. Cells were transfected for 48 h with pCMV6-PSD-DDK-myc plasmids and were then fixed with PFA 4%. After cell permeabilization by triton 15%, PSD was labeled with the anti-DDK antibody and the secondary antibody AF-488 anti-mouse (green). Immunostaining of the mitochondrial network (far red) was obtained with an anti-TOM20 antibody and a secondary one, AF-647 anti-rabbit. (**D**) The fluorescence intensity was measured and illustrated with the line-scan to show the overlaps between Tom20 (far red) and PSD expression (green).

Previous work revealed that PSD genetic inhibition leads to bioenergetics failure, so we tested this possibility in DXR-treated cells. To evaluate the subsequent effect of doxorubicin accumulated in mitochondria on cellular energy production, we measured the mitochondrial respiration and the ATP content of HeLa cells treated with 5 μM doxorubicin for 6 and 24 h (Figure 3A,B). As observed in the genetic models of PSD inhibition [15], the treatment with DXR also induced a significant oxygen consumption rate (OCR) reduction on HeLa cells. 

We verified the localization of PSD within HeLa cells using a tagged version of this enzyme. The immunocytochemistry images revealed a mitochondrial expression of PSD (Figure 3C,D). The co-localization quantification of the enzyme with Tom20, a mitochondrial protein, was estimated with the Pearson index (0.93 ± 0.04, *N* = 22) that was in accordance with an expression within the mitochondrial compartment.

### 2.4. DXR Sensitivity Depends on PSD Dependency for the Mitochondrial Pool of PE

**Figure 4:**
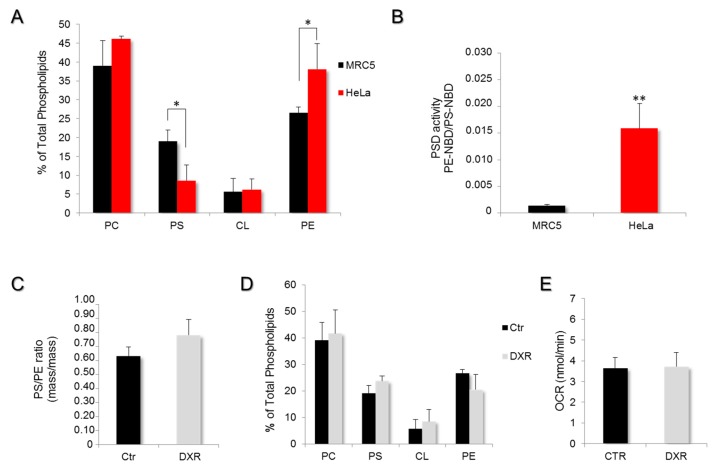
Phosphatidylserine decarboxylase activity is the limiting step in DXR efficiency. (**A**) The phospholipid composition of mitochondrial membranes was determined on other cells, MRC5, by high-performance thin-layer chromatography and compared to that of HeLa. The quantification of PC, PS, CL, and PE was performed by densitometry and expressed as a percentage of total phospholipids (*, *p* < 0.05). (**B**) PSD activity was measured on mitochondrial fractions of MRC5 and compared to those of HeLa cells. The data are expressed in PE-NBD/PS-NBD ratio per minute, as mean values ± SEM of N ≥ 3 experiments (**, *p* < 0.01). (**C**) The PS/PE ratio was determined in MRC5 cells treated or untreated with 5 μM DXR. (**D**) The effect of DXR on the entire mitochondrial membrane composition was assayed. DXR treatment was unable to induce a mitochondrial membrane remodeling within MCR5 cells. (**E**) The mitochondrial respiration was measured in MRC5 cells treated for 6 h with 5 μM DXR, and endogenous rates of respiration were recorded and compared to the control condition. Data are expressed as mean values of oxygen consumption rates (OCR) ± SEM, *N* = 5 experiments.

To investigate the importance of the mitochondrial-membrane destabilizing effect of DXR on cell physiology, we compared HeLa cancer cells and MRC5 non-cancer cells. In the two models, the phospholipid composition of mitochondrial membranes was determined by high-performance thin-layer chromatography and quantified by densitometry (Figure 4A). The MRC5 presented a higher content in PS and a lower proportion of PE when compared to the mitochondrial membrane composition of HeLa cells, suggesting a lowered PSD activity in MRC5. The mass/mass ratio PS/PE was significantly lower (*p* < 0.001) in mitochondrial membranes of HeLa (0.23 ± 0.13) as compared to MRC5 cells (0.63 ± 0.16), indicating a faster conversion of PS to PE in HeLa cells. To confirm, mitochondria isolated from MRC5 cells were incubated with a fluorescent analog of phosphatidylserine (PS-NBD), and the rate of production of phosphatidylethanolamine-NBD (PE-NBD) was followed for one hour. The results were expressed as variations in the ratio PE-NBD/PS-NBD as a function of time (Figure 4B). The data analysis revealed a higher conversion rate of PS-NBD to PE-NBD in the mitochondria of HeLa cells (factor of 8.6 ± 2.6; *p* < 0.01) as compared to MRC5. Therefore, the PE pool that constitutes the mitochondrial membrane of MRC5 cells is provided by the Kennedy pathway instead of PSD. To evaluate the impact of DXR on mitochondrial membrane composition, we treated these MRC5 cells with DXR 5 μM for 72 h prior to the isolation of mitochondria and the analysis of their membrane phospholipid composition (Figure 4C). The results revealed no significant effect on MRC5 (0.74 ± 0.23; *p* > 0.2), while the PS/PE ratio of HeLa cells was modified by DXR (Figure 2B). In fact, the phospholipid composition of mitochondrial membranes in MRC5 remained the same after DXR treatment (Figure 4D). 

To verify whether the absence of the DXR effect on the mitochondrial membrane was due to an artifact in the drug incorporation, this criterion was quantified by fluorimetry after 10 min of incubation. The measurement revealed a similar drug content inside MRC5 and HeLa (Figure S1A). Moreover, to exclude a possible extrusion of DXR outside the cells, the multidrug resistance protein 1 (MDR-1) expression was quantified by RT-qPCR. As expected for a cancer cell line, HeLa cells presented a higher expression level of MDR-1 as compared to MRC5 (Figure S1B). Taken together, these data clearly indicate that the particular effect on cell viability of DXR cannot be explained by differences in drug incorporation, nor by a drug extrusion process.

To evaluate the subsequent effect of doxorubicin accumulated in mitochondria on energy production, the mitochondrial respiration was also measured on MRC5 cells treated with 5 μM doxorubicin for 6 h. The endogenous oxygen consumption rate (OCR) measured on MRC5 showed no DXR effect (Figure 4E).

Regarding the impact on energy, doxorubicin triggered a decrease in the cellular ATP content of HeLa cells (*p* < 0.05) after 6 h of incubation with 5 and 10 μM (86.69% ± 2.88% of control and 82.16% ± 3.66%, respectively) (Figure S2A). This reduction in ATP synthesis was not explained by a direct inhibitory effect of DXR on mitochondrial respiration, because of the fact that OCR measurements after 30 min of treatment with 10 μM DXR revealed no change (data not shown). In MRC5, doxorubicin treatments induced no change in cellular ATP content after 6 h (Figure S2B). Thus, the decrease in ATP content observed in HeLa, under the condition of DXR treatment, is consecutive to the remodeling of the mitochondrial membrane composition.

**Figure 5:**
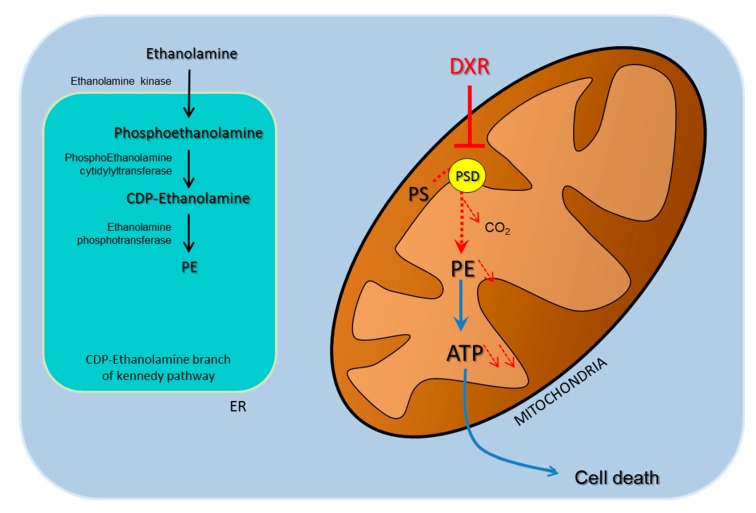
Model of doxorubicin action’s specificity. DXR inhibits PSD activity, alters mitochondrial membrane composition, and triggers impairments in the oxidative phosphorylation (OXPHOS) system that will lead to apoptosis on cells that rely on the PSD pathway as a primary source for the mitochondrial pool of PE.

This led us to propose a model whereby doxorubicin inhibits the first energy metabolism via the alteration of the mitochondrial membrane composition and triggers a reduction in cell viability specifically in HeLa cells (Figure 5). The reduction in PE within the mitochondrial membrane followed by PSD inhibition cannot be compensated by other metabolic pathways such as the CDP-ethanolamine branch of the Kennedy pathway. Then, the oxidative phosphorylation (OXPHOS) system is less efficient. In this model, the reduction of ATP synthesis triggered by DXR will participate until cell death induction and could also decrease the ability of HeLa cells to perform DXR detoxification by the ATP-consuming drug resistance system.

## 3. Discussion

Cancer cells are adapted to rapid growth. Multiple studies revealed changes at the level of cellular energy metabolism with the modulation of the balance between glycolysis and oxidative phosphorylation (OXPHOS) and, moreover, the deviation of metabolic intermediates toward the production of phospholipids, among others [17,18,19,20]. Thus, similar changes could occur at the level of mitochondrial membrane metabolism to accommodate the intense de novo fatty acid biosynthesis reported in most cancer cells [21]. Previous studies reported differences in the mitochondrial membrane lipid composition of cancer cells as compared to non-cancer cells [22]. The differences in composition were explained by changes in the activity of enzymes involved in lipid metabolism, as shown for the serine palmitoyl transferase in Morris hepatoma [23]. Later, biochemical studies performed in rats or mice carrying a tumor also revealed changes in mitochondrial membrane composition, raising the question of the impact of these modifications for tumor biology and survival [24,25]. 

Mitochondria play a significant role in membrane metabolism as they host the key enzyme PSD, which produces a major pool of PE through PS decarboxylation. Previous studies reported an inhibitory effect of Adriamycin on the decarboxylation of PS [26]. In addition, adriamycin is a compound also used in chemotherapy under the name of doxorubicin. However, this particular effect has never been considered in the context of cancer, as doxorubicin’s mode of action. Here, we hypothesized that DXR may have a specificity of action on cell viability due to its effect on PSD-dependent cells, an enzyme implicated in phospholipid metabolism. 

Accordingly, we observed a difference in mitochondrial membrane phospholipid composition characterized by a lower PS/PE ratio in HeLa when compared to MRC5. This difference in membrane composition could be explained by a higher expression level of PSD and/or a higher enzyme activity. In our study, the low PS/PE ratio observed in mitochondrial membranes of HeLa cells was perceptive on mitochondrial fraction. This finding is in accordance with the study of Steenbergen et al., showing that mitochondrial PSD is a major source of PE production for the entire cell [15]. 

Yet, little data are available on the sensitivity of these different tumors to DXR, which does not permit the hypothetic link between PSD expression and sensitivity to doxorubicin to be established. 

In our study, mitochondria isolated from HeLa or MRC5 retained the same total content of PS-NBD imported in the organelle after 3 h of incubation, so no difference could be attributed to the transport rate, in contrast to the study of Voelker et al. [27]. We observed that PSD activity is strongly inhibited by doxorubicin in HeLa cells and isolated mitochondria, while it remained unaffected in MRC5. Previous studies showed a tight binding of DXR with cardiolipin in the mitochondrial matrix face in rat liver and heart mitochondria [11,28]. In our study, the cardiolipin content of HeLa cells was twice that measured in MRC5 cells, so more DXR could bind to the mitochondrial membranes of HeLa cells, although the incorporation of DXR in the two cell types clearly indicated no difference in the accumulation of the drug after 10 min of incubation. 

Mitochondrial ATP synthesis was reduced in HeLa after 24 h of incubation with DXR. This energy crisis occurred in HeLa cells concomitantly with the modification of mitochondrial membrane composition. Moreover, we observed no direct effect of DXR on the mitochondrial respiration of HeLa cells (30 min of incubation), which suggests that a period of 6 h was needed for DXR to inhibit oxidative phosphorylation. Likewise, in another study, important changes were observed in the expression of mitochondrial proteins after the incubation with DXR [29,30]. Such an up-regulation of energy genes related to glycolysis and the Krebs cycle frequently occurs in situations of pharmacological or pathological inhibition of the OXPHOS system [31,32,33], as observed in HeLa cells treated with DXR. These observations suggest an important role for mitochondrial PSD in the regulation of energy production. Tasseva et al. showed that PE deficiency consecutive to PSD expression inhibition triggered impairments in the mitochondrial structure responsible for the rise in mitochondrial membrane potential and the decrease in ATP production [12]. Another study on the disruption of PSD activity in transgenic mice revealed the alteration of mitochondrial energy production, explained by the alteration of mitochondrial membrane composition and structure [15]. The constitutive feature in mitochondrial membrane composition observed in HeLa cells, such as the rapid conversion of PS to PE, could explain the stronger sensitivity of cancer cells to DXR as compared to non-cancer cell lines. The limiting step in PSD pathway is PS transport from the ER to the mitochondrial compartment. Furthermore, this translocation is ATP-dependent. Then, the DXR impact on energy production can amplify PSD inhibition as a vicious circle, by blocking PS transport to the mitochondria.

Moreover, the inhibition of oxidative phosphorylation by DXR in HeLa cells could impair the detoxification mechanisms that require large amounts of ATP [34]. MRC5 cells were characterized by a lack of PSD sensitivity to DXR, indicating a weak involvement of the enzyme in PE synthesis for the mitochondrial membrane, which remained the same after the drug treatment. The same amount of PE within the mitochondrial membrane protects the OXPHOS system. Therefore, the mitochondrial respiration and the ATP content are unaffected.

Overall, the mitochondrial pool of PE could represent a molecular marker of DXR efficiency in chemotherapy, more precisely, in cases of PSD dependence. First, cancer cells that rely on PSD activity, contrary to the normal cells, should be discriminated. Then, this approach may significantly reduce the side effects of the drug treatment such as cardiotoxicity. In addition, other therapeutic alternatives may be used to identify compound inhibitors of PSD activity [35], such as their specific ability to inhibit cancer cell bioenergetics and proliferative activity. Taken together, our findings demonstrate, for the first time, a clear effect of DXR on cells that rely on PSD for the mitochondrial pool of PE synthesis. 

## 4. Materials and Methods

### 4.1. Chemicals

All the reagents were purchased from Sigma-Aldrich (St Louis, MO, USA), with the exception of the ATP Bioluminescence Assay Kit HS II (Roche, Bâle, Switzerland) and PS-NBD (Avanti Polar Lipids, Alabaster, AL, USA). 

### 4.2. Cell Types and Culture Conditions

HeLa cells and MRC5 were purchased from ATCC (Manassas, VA, USA). They were grown in Dulbecco’s Modified Eagle Media (DMEM) from GIBCO (Thermo Fisher Scientific, Waltham, MA, USA), containing 25 mM glucose, supplemented with 10% fetal calf serum, 100 U/mL penicillin, and 100 U/mL of streptomycin. The cells were kept in 5% CO_2_ at 37 °C. For bioenergetics measurements and the phospholipid composition of mitochondria and cellular membranes analysis, cells were harvested during the exponential phase of growth at 80% of confluency. DXR treatment was performed by incubating the cells for 6, 24, or 72 h with different concentrations of this drug, as specified. For the cell enumeration, cells were seeded in a 12- or 24-well plate at a concentration of 100,000 or 10,000 cells per well. The following day, DXR was added into wells at 5 or 10 μM. After 24 or 72 h of incubation, the cells were counted in the presence of trypan blue in a Malassez hemocytometer.

### 4.3. Mitochondrial Isolation

Mitochondrial preparation was performed using digitonin according to the procedure described in Trounce et al., with modifications [36]. Cells were grown in 175 cm^2^ flasks, trypsinized (0.05%), centrifugated (1000 *g*, 10 min), and re-suspended in 1 mL of ice-cold buffer I (210 mM mannitol, 70 mM sucrose, 5 mM HEPES, 1 mM EGTA, 0.5% BSA). Digitonin was added to reach a final concentration of 1 mM, and cells were incubated for 15 min on ice. Cell permeabilization was verified under the microscope, using trypan blue. A centrifugation (625 *g*, 5 min) was performed to remove digitonin, and the pellet was re-suspended in 10 mL of buffer I for homogenization in a glass potter on ice (20 strikes, gently). Again, cell membrane disruption was verified under the microscope. A centrifugation was performed (625 *g*, 5 min), and the supernatant was kept on ice and resubmitted to centrifugation (10,000 *g*, 20 min). The mitochondrial pellet was re-suspended in buffer I. The typical yield allowed around 1 mg of mitochondrial proteins per million cells to be obtained. 

### 4.4. Lipid Composition of Mitochondrial and Cellular Membranes

The phospholipid composition was determined either on entire cells or on isolated mitochondria. For the mitochondrial membranes, the organelle isolation was performed as described above and sub-mitochondrial membranes were purified according to Ardail et al., with modifications [37]. The protein amount of the mitochondrial fraction was determined with a Bradford protein assay using bovine serum albumin as standard [38]. Lipids were extracted and purified according to Bligh and Dyer [39]. Phospholipids were separated by mono-dimensional HPTLC (high-performance thin-layer chromatography) using the solvent system described by Vitiello and Zanetta [40]. They were detected by spraying the plates with a solution of 0.1% (*w*/*v*) primuline in 80% acetone and imaged under UV light. Lipids were then quantified from HPTLC plates by densitometric scanning after copper sulphate staining from at least three independent biological samples and expressed as a percentage of total phospholipids [41].

### 4.5. Cell Viability Assay

The cytotoxicity of DXR was evaluated on cells by using the neutral red assay, as detailed by Borenfreund and Puerner [42,43] and as previously used in Nouette-Gaulain et al. [42,43]. After 24 h of incubation with different DXR concentrations, absorbance was measured in a microplate reader spectrophotometer (MP96, SAFAS, Monaco) at a wavelength of 540 nm. The cell viability data were expressed as a percentage of untreated cell absorbance.

### 4.6. PSD Activity Fluorimetric Assay

Phosphatidylserine decarboxylase activity was measured on isolated mitochondria or on entire cells. For the in vitro assay, mitochondria were isolated from the different cell types, as described above, and a fraction (150 μg of proteins) was re-suspended in 200 μL of buffer I and incubated at 37 °C, with 10 μg of the fluorescent analog of phosphatidylserine (PS-NBD, Avanti Polar Lipids). This phospholipid was converted to phosphatidylethanolamine-NBD (PE-NBD) by PSD, and every 5 min, an aliquot was frozen and analyzed for lipid composition, as described previously. After the migration of each sample on the plates, PS-NBD and PE-NBD were identified according to the standards. The fluorescence intensities were measured by scanning the plates (λex: 460 nm; λem: 535 nm). The rate of PE-NBD formation was calculated in the initial phase of the reaction by using a linear regression (Excel). The results of the PSD specific activity were expressed as the ratio PE-NBD/PS-NBD/min/μg of protein. The data are presented as the mean value ± SD of three different experiments.

### 4.7. ATP Measurements and Cell Respiration

To assay their ATP content, the cells were seeded in a 96-well white plate with a clear bottom at 8000 cells per well. The following day, cells were incubated in the presence or absence of 5 μM doxorubicin for 6 or 24 h. The intracellular ATP content was measured by using the bioluminescent ATP Kit HS II (Roche Applied Science, Bâle, Switzerland). Then, 50 μL of lysis buffer were added to each well and the cells were incubated for 5 min at RT to release the intracellular ATP. Next, 50 μL of luciferase were injected in a bioluminescence multiplate reader (Luminoskan Microplate Luminometer, Thermo Fisher Scientific, Waltham, MA, USA) and, after 1 s of incubation, the bioluminescence was read (10 s integration time) in relative light units (RLU). Standardization was performed with known quantities of standard ATP in the same conditions. 

Mitochondrial oxygen consumption was monitored at 37 °C in a 1 mL thermostatically controlled chamber, equipped with a Clark oxygen electrode (Hansatech, Pentney, UK). The routine (endogenous) respiration was measured on 2 million cells in DMEM treated or untreated with 5 μM of DXR.

### 4.8. Fluorescence Microscopy

Cells were grown in high-glucose DMEM on a 4-well LAB-TEK (Dutscher, Mérignac, France). The mitochondrial network was stained with the Mitotracker Green dye (Molecular Probes, Eugene, OR, USA), used at 100 nM for 30 min and 5 μM of DXR at 37 °C for 10 min. The cells were visualized by fluorescence microscopy. The fluorescence intensity was measured in the green (ex 490 nm/em 516 nm) and the red (ex 470 nm/em 585 nm) by using appropriate filters. For these observations, a Zeiss microscope (AxioVision, Carl Zeiss, Oberkochen, Germany) was used, with a 63× oil objective. DXR accumulation in the mitochondrial compartment was estimated with Carl Zeiss software. Pearson’s correlation coefficients were determined. 

### 4.9. RT-PCR on Cultured Cells

RNA isolation was performed on the different cellular pellets with the RNeasy^®^ Mini kit (Qiagen, Hilden, Germany) according to the manufacturer’s instructions. In addition, 1 μg of the total RNA was reverse-transcribed into cDNA using the reverse transcription system (Promega, Charbonnières-les-bains, France,) at 42 °C for 120 min, followed by a step of reverse transcriptase inactivation at 70 °C for 15 min. Real-time quantitative PCR was performed with an iCycler (Bio-Rad Hercules, CA, USA). In brief, triplicate PCR reactions were assembled in 0.1 mL strip tubes containing cDNA from 50 ng of total RNA, iQ^TM^ SYBR^®^ Green Supermix (Bio-Rad) containing iTaq DNA polymerase and 0.4 mM of each dNTP and 6.5 μM of each of the appropriate primer. The PCR was performed under the following conditions: Denaturation at 95 °C for 30 s, annealing at 60 °C for 30 s, and extension at 70 °C for 30 s for 30–40 cycles. PCR negative controls were systematically made using water instead of the cDNA sample. All specific primers were ordered from Sigma-Aldrich (Saint-Quentin Fallavier, France). Primers, sense and antisense, were for MDR1 (forward 5’-TTGGCCATCAGTCCTGTTCTT-3’ and reverse 5’-TGTCCTCCAAATGCAATCACA-3’). The RT-PCR expression of the target genes was then presented as an arbitrary unit and normalized to the RPLP0 (forward 5’-GGCGACCTGGAAGTCCAACT-3’ and reverse 5’-CCATCAGCACCACAGCCTTC-3’) and Gus B (forward 5’-GGAGAGCTCATTTGGAATTTTGCCG-3’ and reverse 5’- TGGCTACTGAGTGGGGATACCTGG-3’) as references.

### 4.10. Western Blot

Cell lysis was performed by using RIPA buffer for one hour on ice. After protein quantity determination with a BCA kit (Pierce, Thermo Fisher Scientific, Waltham, MA, USA), the samples were diluted into an SDS-PAGE sample buffer (Bio-Rad) with 4% *β*-mercaptoethanol. The samples were loaded in a 10% SDS polyacrylamide gradient mini-gel (Bio-Rad) and separated by electrophoresis at 150 V. After the migration, proteins were transferred to 0.2 μm polyvinylidene difluoride (PVDF) membranes, with a Turbo Trans-blot system. Membranes were blocked for one hour in 5% milk-PBS containing 0.05% Tween, and incubated overnight with primary antibodies. Antibodies against phosphatidylserine decarboxylase and β-actin were purchased from Santa Cruz Biotechnology (sc-86197) (Dallas, TX, USA) and Sigma Aldrich, respectively. After three washes with PBS-0.05% Tween 20, the membranes were incubated for 1 h with horseradish peroxidase-conjugated goat anti-rabbit (Bio-Rad), diluted in 5% milk-PBS. This secondary antibody was detected in a Chemidoc (Bio-Rad) using the chemiluminescent SuperSignal™ West Pico PLUS Chemiluminescent Substrate (Pierce). The signal was quantified by densitometric analysis using Image J (NIH, USA) software.

### 4.11. Statistical Analysis

All the data presented in this study correspond to the mean value of N experiments ± SD or SEM when specified, with N ≥ 3. A comparison of the data sets obtained was performed with the Student’s t test or the appropriate statistical analysis, using Excel Software (Microsoft, Redmond, WA, USA) and GraphPad Prism software (GraphPad Software, San Diego, CA, USA). Two sets of data were considered statistically different when *p* < 0.05.

## Supplementary Materials

Supplementary materials can be found at https://www.mdpi.com/1422-0067/21/4/1317/s1.
