# Supplementary material for: Doxorubicin Inhibits Phosphatidylserine Decarboxylase and Modifies Mitochondrial Membrane Composition in HeLa Cells
Source: Int J Mol Sci. 2020 Feb 15;21(4):1317. doi: 10.3390/ijms21041317 (PMC7072979; doi:10.3390/ijms21041317)
Supplement: Supplementary file 1 [file ijms-21-01317-s001.pdf]

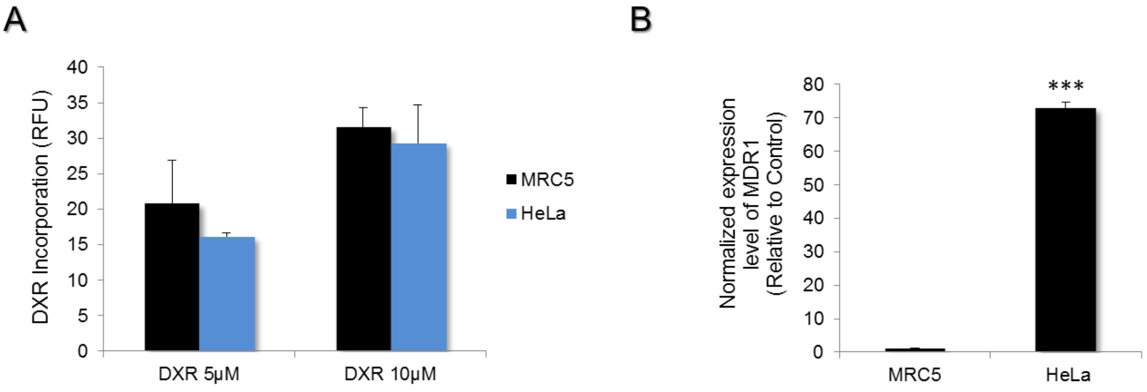

Supplemental S1. DXR accumulation within the different cell lines is identical. (**A**) The drug uptake was measured in relative fluorescence units (RFU), after 10 min of treatment with 5 or 10 µM of DXR. The results are presented as mean values of 3 different experiments ± SD. (**B**) The expression of multidrug resistance protein 1 (MDR1) was analyzed by RT-qPCR. The bars represent expression level ± SEM, with ***, $P < 0.0001$.

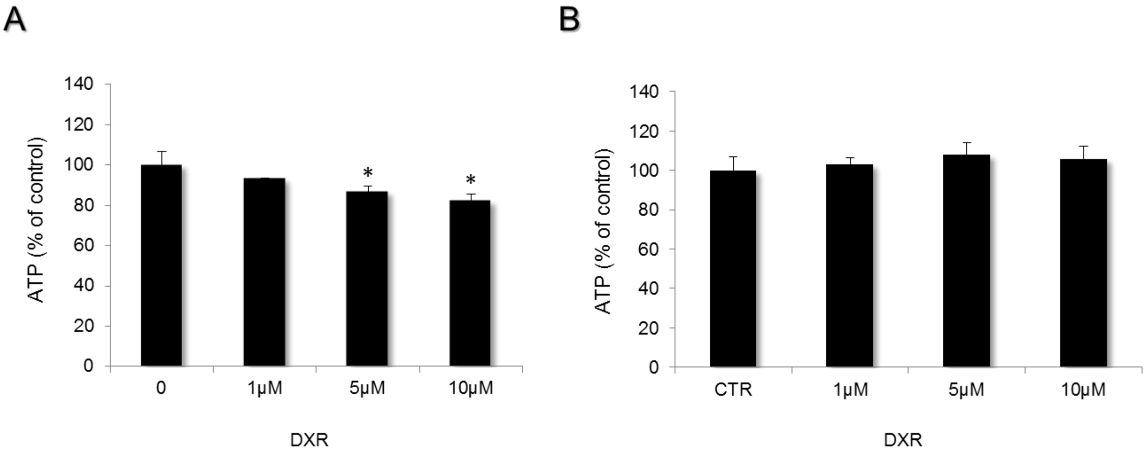

Figure S2. DXR impairs bioenergetics of PSD-dependent cells. The ATP content was measured in (**A**) HeLa and (**B**) MRC5 cells after 6 h of incubation with DXR at 1, 5, or 10 µM. The data are expressed in percentage of control, as untreated cells. The bars represent mean values ± SD with *, $P < 0.05$.
